# A case of spontaneous lumbar artery rupture presenting with retroperitoneal hematoma and extensive subcutaneous hemorrhage

**DOI:** 10.1093/omcr/omae008

**Published:** 2024-03-25

**Authors:** Junki Morino, Keiji Hirai, Yoshiyuki Morishita

**Affiliations:** Division of Nephrology, First Department of Integrated Medicine, Saitama Medical Center, Jichi Medical University, Saitama-ken, Japan; Division of Nephrology, First Department of Integrated Medicine, Saitama Medical Center, Jichi Medical University, Saitama-ken, Japan; Division of Nephrology, First Department of Integrated Medicine, Saitama Medical Center, Jichi Medical University, Saitama-ken, Japan

**Figure 1:**
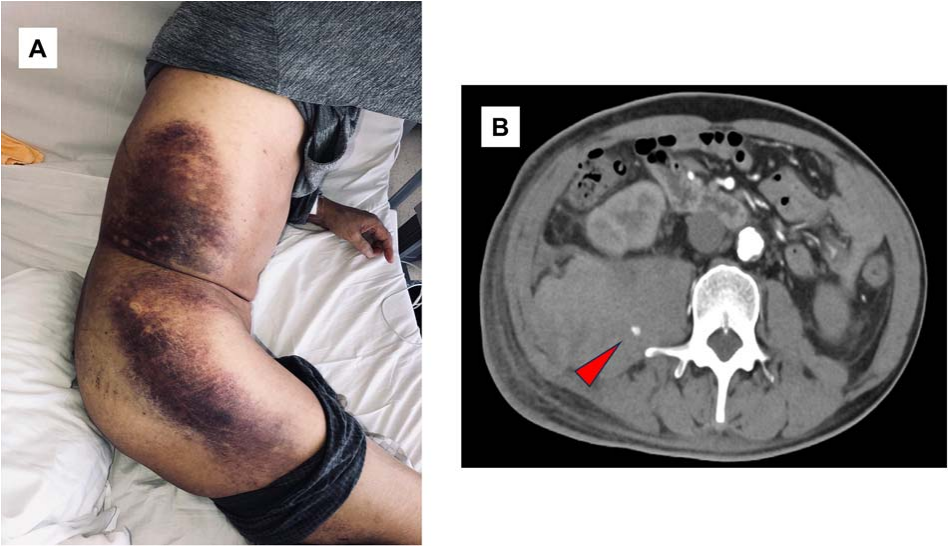
(**A**) Clinical photograph obtained on hospital day 8. Subcutaneous hemorrhage on the right flank and buttock. (**B**) Contrast-enhanced computed tomography scan obtained. Axial computed tomography scan showing right-sided retroperitoneal hematoma and lumbar artery rupture with extravasation of contrast medium (arrowhead).

A 63-year-old man with advanced diabetic nephropathy and old cerebral infarction was referred to us with a 2-week history of fatigue and difficulty moving. He was on treatment with antidiabetics, and aspirin. Physical examination showed no subcutaneous hemorrhage. Blood pressure was 139/76 mmHg. Laboratory investigations showed renal dysfunction and hyperkalemia (creatinine 18.0 mg/dl, blood urea nitrogen 111 mg/dl, potassium 6.6 mEq/l). He was started on hemodialysis immediately. On hospital day 2, he developed right-sided back pain. On the following day, his hemoglobin level decreased from 12.0 to 5.5 g/dl and blood pressure to 104/59 mmHg. Upper and lower gastrointestinal endoscopy didn’t detect a bleeding source. His anemia didn’t improve despite transfusion of 8 units of red cells. On hospital day 8, extensive subcutaneous hemorrhage was observed on his right flank, buttock, and thigh (Fig. 1A). Contrast-enhanced computed tomography (CT) revealed a ruptured right lumbar artery and massive retroperitoneal hematoma with extravasation of contrast medium (Fig. 1B). The diagnosis was spontaneous lumbar artery rupture. Transcatheter arterial embolization was performed and improved the hemoglobin level from 5.5 to 8.9 g/dl. His symptoms improved, and he was discharged on hospital day 21.

Retroperitoneal hematoma with lumbar artery rupture is reportedly a rare condition caused by trauma, malignancy, aortic aneurysm but may also be iatrogenic or spontaneous [1]. Old age, end-stage kidney disease, hemodialysis, and anticoagulant/antiplatelet therapy are risk factors for spontaneous lumbar artery rupture [2]. Our patient had severe renal dysfunction and had received antiplatelet therapy, which are reportedly risk factors [2]. Clinical symptoms of lumbar artery rupture include flank pain, hypotension, and anemia [3]. Enhanced CT is useful for diagnosis. Our patient developed massive retroperitoneal hematoma and extensive subcutaneous bleeding with persistent anemia and hypotension. Transcatheter arterial embolization and surgical hemostasis have been recommended for treatment [2]. Transcatheter therapy was administered in our patient and hemostasis was achieved. Physicians should be aware of lumbar artery rupture when subcutaneous hemorrhage on flank, buttock, and thigh was observed.
